# Barriers and facilitators to the implementation of a school-based physical activity policy in Canada: application of the theoretical domains framework

**DOI:** 10.1186/s12889-017-4846-y

**Published:** 2017-10-23

**Authors:** Katie A. Weatherson, Rhyann McKay, Heather L. Gainforth, Mary E. Jung

**Affiliations:** 1 School of Health and Exercise Sciences | Faculty of Health and Social Development, The University of British Columbia Okanagan, ART 360– 1147 Research Road, Kelowna, BC V1V 1V7 Canada; 2 School of Health and Exercise Sciences Faculty of Health and Social Development, The University of British Columbia Okanagan, ART 129– 1147 Research Road, Kelowna, BC V1V 1V7 Canada; 3 School of Health and Exercise Sciences Faculty of Health and Social Development, The University of British Columbia Okanagan, RHS 119­ 3333 University Way, Kelowna, BC V1V 1V7 Canada

**Keywords:** School, Physical activity, Policy, Implementation, Barriers, Facilitators, Theoretical domains framework

## Abstract

**Background:**

In British Columbia Canada, a Daily Physical Activity (DPA) policy was mandated that requires elementary school teachers to provide students with opportunities to achieve 30 min of physical activity during the school day. However, the implementation of school-based physical activity policies is influenced by many factors. A theoretical examination of the factors that impede and enhance teachers’ implementation of physical activity policies is necessary in order to develop strategies to improve policy practice and achieve desired outcomes. This study used the Theoretical Domains Framework (TDF) to understand teachers’ barriers and facilitators to the implementation of the DPA policy in one school district. Additionally, barriers and facilitators were examined and compared according to how the teacher implemented the DPA policy during the instructional school day.

**Methods:**

Interviews were conducted with thirteen teachers and transcribed verbatim. One researcher performed barrier and facilitator extraction, with double extraction occurring across a third of the interview transcripts by a second researcher. A deductive and inductive analytical approach in a two-stage process was employed whereby barriers and facilitators were deductively coded using TDF domains (content analysis) and analyzed for sub-themes within each domain. Two researchers performed coding.

**Results:**

A total of 832 items were extracted from the interview transcripts. Some items were coded into multiple TDF domains, resulting in a total of 1422 observations. The most commonly coded TDF domains accounting for 75% of the total were *Environmental context and resources* (*ECR*; *n* = 250), *Beliefs about consequences* (*n* = 225), *Social influences* (*n* = 193), *Knowledge* (*n* = 100), and *Intentions* (*n* = 88). Teachers who implemented DPA during instructional time differed from those who relied on non-instructional time in relation to *Goals*, *Behavioural regulation*, *Social/professional role and identity*, *Beliefs about Consequences*. Forty-one qualitative sub-themes were identified across the fourteen domains and exemplary quotes were highlighted.

**Conclusions:**

Teachers identified barriers and facilitators relating to all TDF domains, with *ECR*, *Beliefs about consequences*, *Social influences*, *Knowledge* and *Intentions* being the most often discussed influencers of DPA policy implementation. Use of the TDF to understand the implementation factors can assist with the systematic development of future interventions to improve implementation.

**Electronic supplementary material:**

The online version of this article (10.1186/s12889-017-4846-y) contains supplementary material, which is available to authorized users.

## Background

Levels of physical activity are assiduously low among children and youth in Canada [1] and worldwide [2], and have in part contributed to the increased rates of childhood overweight and obesity and associated chronic diseases (e.g., cardiovascular diseases and type 2 diabetes) [3, 4]. Establishing healthy lifestyle behaviours, like physical activity, is imperative during childhood, as these behaviours can extend across the life span [5] and have long-term health implications (e.g., prevention of weight gain/obesity, type 2 diabetes, coronary heart disease, dementia, Alzheimer’s disease) [6]. Consequently, public health governing bodies have prioritized strategies and interventions to combat children’s physical inactivity and obesity crisis globally [7, 8] and within Canada [9]. In Canada, schools are often the target of such initiatives as they represent an environment through which to reach a large and diverse population of youth, who spend a majority of their waking time in school [10, 11].

Several provinces in Canada have adopted daily physical activity policies aimed at increasing children’s physical activity specifically during the school day [12–14]. In British Columbia, the Ministry of Education mandated a Daily Physical Activity (DPA) policy in 2008 (revised in 2011) requiring elementary schools to provide at least 30 min of DPA as part of the educational program for children in grades Kindergarten to seven [12]. Specifically, the DPA policy requires elementary students to achieve 30 min of physical activity at school on days with no physical education.^1^ This requirement includes any activities that help build endurance, strength and flexibility (e.g., walking, running, push-ups, stretching) and that are conducted during instructional (i.e. within-class) or non-instructional (i.e. recess or lunch break) time.

In order to improve the success of such policies, it is advised that policy evaluation occur at the outset and continues on an ongoing basis [15]; however, minimal research in Canada has examined the process of how DPA policy plans are translated into practice (i.e., implementation) and there is currently no research examining the effectiveness of the DPA policy in BC schools [16]. Central to understanding the implementation process is a comprehensive and theoretical examination of the numerous factors that can impede (i.e., barriers) or enhance (i.e., facilitators) the successful implementation of physical activity policies at a local school-level. While some research has identified barriers and facilitators to DPA implementation in Canada [17–21], theory is rarely used to guide our understanding of these factors [22]. Behaviour change theories postulate the psychological and environmental constructs that affect behaviour by specifying mechanisms of change. Within the school context, utilizing a theoretical approach allows researchers to systematically identify the potentially malleable factors affecting teacher’s implementation of the policy and to prioritize and develop strategies through which to target these key factors to improve policy practice and achieve desired outcomes. For this reason, this study moves beyond the simple identification of barriers and facilitators to DPA policy implementation by descriptively linking these factors to pathways of behaviour change in order to enhance implementation practices [23].

To achieve this aim, this study uses the Theoretical Domains Framework (TDF). The TDF, developed and validated by Michie and colleagues, is an integrative framework that synthesizes over eighty constructs across thirty-three psychological theories in order to understand influences on behaviour more broadly [24, 25]. Specifically, the TDF is organized into 14 categories, called domains, to categorize the potential range of behavioural and organizational factors that influence implementation outcomes [26]. Domains that address behavioural factors include: Knowledge, Skills, Memory, attention and decision processes, Behavioural regulation, Social/professional role and identity, Beliefs about capabilities, Optimism, Beliefs about consequences, Intentions, Goals, Reinforcement, and Emotion. Domains that address organizational factors include: Environmental context and resources, and Social influences (TDF domain definitions are provided in Additional file 1).

The TDF has been successfully applied in many settings to identify influences on a variety of behaviours [27]. There are many individual, environmental and social-cultural factors that influence the successful implementation of policies in schools. For example, some of the factors shown to influence implementation include: leadership and support, resource support, communication/shared decision-making, and individual self-efficacy/skills [28]. Therefore, a framework that can capture these influences operating at different levels is warranted.

More broadly, the TDF is a refined version of the Capability Opportunity Motivation-Behaviour (COM-B) model, an evidence-based model supporting that three key sources (i.e., capability, opportunity and motivation) interact to influence behaviour. The COM-B model can be linked to a practical intervention design tool called the Behaviour Change Wheel framework (BCW) [26] to guide researchers in the selection of theory, intervention functions, policy categories, and behaviour change techniques for intervention design and delivery. As a result, the TDF is one of few frameworks linked to a comprehensive method for intervention design.

### Purpose

The purpose of this study was to use the TDF to understand teachers’ barriers and facilitators to the implementation of the Daily Physical Activity policy in British Columbia elementary schools. Additionally, barriers and facilitators were examined and compared according to how the teacher implemented the DPA policy during the school day (provision of DPA during instructional time or only non-instructional time).

## Methods

### Overall design

This study used short surveys and semi-structured interviews to explore the factors (i.e., barriers and facilitators) associated with the implementation of the Daily Physical Activity policy by elementary school teachers in one school district in British Columbia. A content analysis was conducted using the TDF and overarching themes were identified within each domain. Ethical approval was obtained from the University of British Columbia’s Behavioral Research Ethics Board for research involving humans, and the respective school district. The Consolidated criteria for reporting qualitative research (COREQ) [29] guided reporting of this study (see Additional file 2).

### Framework

The first author used the TDF to construct the semi-structured interview guide [see Additional file 3], which underwent revision by HG who is experienced in application of the TDF and was further refined after piloting the interview with two elementary school teachers. The TDF was then used to categorize the implementation barriers and facilitators and explore emergent themes by domain.

### Sample selection and recruitment

One school district from British Columbia representing over 30 public elementary schools was chosen for convenience and approached to participate in this study. Principals of all elementary schools were emailed an information letter to request time to present the study to their intermediate teachers. The first investigator visited the school and conducted a presentation to the teachers, which consisted of information regarding the researcher’s background and interests and her study purpose and details. Teachers were eligible to participate if they were grades 4, 5 or 6 certified school teachers in publicly funded elementary schools with at least one year of experience teaching at an elementary school level, and were currently teaching in the 2015–2016 school year. In total, principals from 13 elementary schools (42% response rate) provided approval for their school to participate, with 33 (of 40) teachers from 11 of these schools (83% response rate) providing written consent to participate in a survey and potentially participate in the interview. The short survey instrument consisted of questions relating to the teacher’s DPA implementation approaches and basic demographic information and was used as a device to assist in selecting and describing the interview sample. Based on survey responses, maximum variation sampling [30] was used to recruit teachers to be interviewed to ensure representation across teacher-reported implementation approaches, which continued until data saturation was reached [31]. In total, twelve interviews were conducted with thirteen teachers (4 male, 9 female), who were aged 30–60 years (*M* = 44.69, *SD* = 10.33) and varied in teaching experience from 5 to 34 years (*M* = 15.69, *SD* = 9.31). Of those teachers who were interviewed, one teacher taught grade 4, three teachers taught grade 4/5, two teachers taught grade 5, five teachers taught grade 5/6 and two teachers taught grade 6. Ten teachers reported implementing DPA by providing additional opportunities to be active during instructional time (instructional implementers), while three teachers were classified as non-instructional implementers because they relied on students being active during non-instructional lunch and recess breaks.

### Data collection

The first investigator conducted twelve semi-structured interviews with 13 teachers between February and April 2016, at a time and location convenient to each teacher (e.g., classroom, coffee shop). All interviews were conducted individually except for one interview, which included two grade 6 teachers from one school. The latter was done because these teachers share a formal platooning schedule (i.e., complete curriculum together within two classrooms), thus reporting the same DPA implementation approach. Each interview was between 31 and 64 min in duration (*M* = 52.25, *SD* = 9.65) and consisted of a broad open-ended question (i.e., “Are there any factors that affect if or how you implement DPA in your classroom during class time? If so, what?”) to elicit perceived barriers and facilitators impacting the implementation of the DPA policy by teachers. Probing questions were used to clarify domain-specific content if the participant had mentioned factors that appeared to fit within a certain domain (see Interview guide in Additional file 3 for more information). This approach was used to minimize leading questions. Field notes were taken by the interviewer during the interview to ensure each relevant domain was discussed further. Verbal consent was obtained from each participant to audio-record the interview and participants received a monetary reimbursement ($20) for their participation.

### Data extraction and analysis

Digital recordings were transcribed verbatim directly into NVivo Version 11 [32] by the first author and two research assistants. Interview transcripts were checked for accuracy by the interviewer; however, the transcripts were not returned to participants for comment. We employed a deductive and inductive analytical approach in a two-stage process whereby extracted barriers and facilitators were 1) deductively coded using pre-existing domains (content analysis based on TDF), and 2) analyzed for emergent themes within each domain. This analysis procedure was chosen because it provides a simple method for summarizing findings in the context of focused evaluation questions, while allowing exploration of unanticipated factors associated with implementation, and is commonly used in health research [33, 34] and TDF analyses [35–38].

### Barrier and facilitator extraction

Barrier and facilitator extraction was performed by the first author, with double extraction occurring across 33% (*n* = 4) of the transcripts by RM to ensure the trustworthiness of the data extraction and coding. Coders read through each interview transcript line-by-line, highlighting and coding the text to ‘Barrier’ or ‘Facilitator’ nodes (containers), operationalized as any factor, characteristic, view or belief that either impeded or enabled implementation of DPA by teachers during the instructional school day, respectively. Barriers and facilitators were extracted if the teacher being interviewed commented that the factor affected their own personal implementation of DPA or if they thought it affected other teachers’ implementation of DPA (i.e., shadowed data). Hypothetical barriers and facilitators, characterized as a factor that the teacher perceived (versus experienced/encountered) to be a potential barrier or facilitator to them or other teachers, were not extracted (e.g., belief that specific resources or support would be helpful for implementation without past experience with these resources/support). If a teacher discussed the same barrier/facilitator at different times within the interview, the factor was counted as separate items. Therefore, the total frequency (count and percent) coded to each TDF domain represents the proportion of interview time spent discussing these factors within each domain. Discrepancies in extraction were discussed until a consensus was reached. Agreed upon barriers and facilitators were transferred to an Excel spreadsheet for TDF coding.

### Barrier and facilitator coding

Two researchers independently coded barriers and facilitators from each interview over twelve rounds (each interview was a new round), with the order of each round being selected at random. As we were attempting to understand barriers and facilitators within the school context (and not test the reliability of the TDF), researchers coded in rounds and met to discuss discrepancies after each round. In the first round, identified barriers and facilitators were coded using the TDF domain and definitions as a coding framework (see Additional file 4) [26]. Where coding varied, consensus was achieved through discussion and the coding manual was refined for subsequent coding rounds to facilitate consistency of TDF coding (see 3rd column in Additional file 4). In the case of particularly challenging exerts, expertise was sought from an expert coder who is knowledgeable and experienced in application of the TDF. Coders also made notes and comments on the overall meaning of each exert during each coding round and responses were compared across teacher-reported implementation approach type. The first coder identified main themes from each domain and exemplary quotations for each theme were selected, consistently cross-checking themes to original transcripts. Negative cases were highlighted and used to refine themes that accounted for the majority of cases. To confirm that interpretations were supported by the data, the themes were presented to the second coder and to an additional researcher who was not part of the data collection, extraction and coding for feedback.

### Reliability

Percent agreement was used to show agreement on barrier and facilitator extraction. Percent agreements, Cohen’s Kappa statistic [39] and prevalence-adjusted bias-adjusted Kappa statistic (i.e., PABAK) [40] were used to show agreement between coders on categorizing the barriers and facilitators by TDF domain, for new items coded at each round as well as for the overall total. PABAK represents the Kappa statistic that adjusts for 1) shared bias in the coders use of categories, and 2) the high prevalence of negative agreement (i.e., when both coders agree on non-contributing domains) and was used to account for the high prevalence of not assigning more than one domain to each barrier. Inter-coder agreement values of 0.60–0.79 indicate “substantial” reliability and those above 0.80 are “outstanding” [41].

## Results

### Reliability

The two independent coders extracted a total of 343 barriers/facilitators from four randomly selected interviews and percent agreement across all extraction rounds was 86.3% (see Additional file 5). A total of 900 factors (417 barriers, 483 facilitators) were extracted across the twelve interviews. Upon coding, 68 (26 barriers, 42 facilitators) factors were deemed ineligible (due to being hypothetical or not affecting the targeted behaviour) and removed from the data set (see Additional file 6), leaving a total of 832 items. All items were coded into at least one of the fourteen TDF domains or an ‘*Other*’ category (for items that did not clearly fit into a pre-defined domain). Some items were coded to multiple TDF domains, resulting in a total of 1422 observations. Across all barrier and facilitator coding rounds, the average inter-coder agreement was outstanding (Percent agreement = 59.7%; Kappa = 0.73 ± 0.37; PABAK = 0.91 ± 0.13). Overall reliability improved following refinement of the coding manual (see Additional file 7) and consensus of final codes was reached through discussion, resulting in 1141 final barrier and facilitator codes.

### Implementation barriers and facilitators

Table 1 presents the summary of TDF domains, themes and quotes organized hierarchically by percent frequency for all participants. Accordingly, the most commonly coded TDF domains accounting for 75% of the total barriers and facilitators were *Environmental context and resources* (*ECR*; *n* = 250; 21.9%), *Beliefs about consequences* (*n* = 225; 19.7%), *Social influences* (*n* = 193; 16.9%), *Knowledge* (*n* = 100; 8.8%), and *Intentions* (*n* = 88; 7.7%). Only two items were classified as *Other* (or uncodable), due to a lack of specificity. Additional file 8 outlines the frequency (total count and percent) of barriers/facilitators that were identified across each TDF domain by implementation approach group. Across all participants, more facilitators than barriers were discussed in relation to *Knowledge*, *Behavioural regulation*, *Beliefs about consequences*, *Goals*, and *Social influences* domains. Barriers and facilitators were equally discussed in *Beliefs about capabilities*, *Optimism*, and *Intentions* domains. Non-instructional implementers discussed rarely or not at all factors related to *Memory, attention and decision processes*, *Behavioural regulation*, and *Goals*. These differences are explored more descriptively in the next section.

**Table 1:** Summary of themes and sample quotes/explanations assigned to the theoretical domains and organized by frequency

TDF Domain	Frequency (% total)	Theme	Sample quotes/explanations
Environmental context and resources	21.9	Lack of time due to curricular demands *and* schedule interruptions (B)	“And honestly, in my world, the days fly. And just accomplishing the curriculum is enough in those hours that we’re given with the kids. That’s what I find.” (Non-implementer 1) “Because of what you have to jam into your day. You’ve got to do reading, you’ve got to do writing, you have to do - especially at elementary school - you have to do math. You have music. You have science, socials. You have like, we’re teaching 12 different subjects, right. So, umm there’s days where ya, it’s hard to get that in there. Ya, for sure.” (Implementer 5) “And time, like realistically, like our teaching day is- there is a lot to get through. And there’s - in elementary school there are so many interruptions in the day. So actually like full instructional days, sometimes you just feel- like you always feel like you are racing against time. And this term in particular. You get like pro-days and assemblies and you’re out on workshops, or whatever. So it just becomes too- that time is always your-- and to give up like 30 min, that’s a lot of time. It doesn’t seem like it but in a day, like it’s a lot.” (Implementer 4)
		Resources (ideas or equipment) and administration or training workshops are helpful/sufficient (F) versus not age-appropriate/insufficient (B)	Overall, teachers explained that the resources made available when DPA was first mandated were helpful but have since gone missing or been broken. Some teachers discussed how the resources were silly and not age-appropriate for older students.
		Teachers’ autonomy is decreased (B) versus supported (F)	“I think before it became a report card thing, I think a lot of us were having some sort of break within the day because we know it’s needed. But to kind of have where it’s like well you have to do it- telling someone you have to do something, changes it. I think if you don’t have to do it, sometimes you are more willing to do it. Like today, the concert was voluntary. Well we all showed up to it, right? We’re not stupid. Fifty minutes of you know, taking them out of class and, you know? They can listen to music and get some music enlightenment. But I think the ‘Big Brother method’ doesn’t work well.” (Implementer 8) “Like, I feel supported that they give us the flexibility to do it at any point in the day.” (Implementer 1)
		There are space constraints (B)	“We have space constraints. My kids are very big and so, you know when they’re… they like to move and they like to move big! So, when we do something in the classroom like aerobics, we’ve got desks everywhere and it’s really difficult to do anything where they’re lying down. So that’s definitely… I’d say even more than time, it’s space.” (Implementer 10) “But we don’t have like the carpet areas like the primary’s would have - where you have room that you could do aerobic type stuff or that ActionSchools stuff, or… because of the size of the children. It’s squishy.” (Non-implementer 3)
		It depends on the weather (B,F)	“I definitely think that weather though is a huge factor in the amount that people get because I notice in the Spring time there is way more classes outside doing things and being active. Because in the wintertime, what do you do? Like it’s mucky, it’s snowy, it’s cold. So to get dressed- especially if you have primary kids- and go out, it’s like, it’s a huge job.” (Implementer 4)
		It is harder at an intermediate level (B)	“When the DPA first came out, I tried. You know, we had those, you know, ‘Get Up and Move and Dance.’ And I found it, honestly, I found it easier in primary doing it then I have in intermediate. Because it seemed like the things, the projects that we did were shorter projects and they were shorter chunks of time. And you just had more space in the classroom. And so we did get up and do, you know, impromptu dance parties or, you know, chair aerobics. You know, we did those kinds of things that they brought in to teach us how to do. But when I got to intermediate and the demands became greater, and they do have a longer attention span…so I definitely in intermediate feel the demands of the time more so than I did in primary. It was much easier in primary to do this.” (Non-implementer 2) “I’ve just found with the older kids that sometimes - like there’s definitely kids into the games and stuff and then there’s other kids that they hate that. At the age that they’re at.” (Implementer 3)
Beliefs about consequences	19.7	Takes time out of schedule (B)	“I was just going to say I can’t think of any negative impact other than the fact that it takes away from teaching time- If you are incorporating it outside of the lunch and the recess.” (Implementer 3) “I think some teachers just don’t see the importance of it or feel like they – it’s one more thing they just can’t afford to lose instructional time on.” (Implementer 7)
		Requires extra planning and set up time (B)	“Because already as a teacher you spend so much of your own time during your lunch hour, your prep or after school preparing for like your core subject areas. And then so to prep like for DPA, just- it’s a lot as it is…” (Implementer 4)
		No impact on PA levels (B) versus increases PA levels of those who need it most (F)	“If you look at some of these kids that’s all they do at lunch and recess is play. They come in exhausted because, you know… and the ones that don’t, don’t do it anyway. Like that’s the irony. Like a lot of the kids that don’t run around at recess, probably don’t… they’re not the ones that are running around at DPA either, right?” (Implementer 8) “Yes I would say [students are more active with DPA compared to without]-- and when you asked that question, like I think of particular students. Um because I know that those ones would not move. Like I watch them outside too and they just kind of hang around or sit out there. Like they’re not the ones who play either. So if we don’t do it, for those ones, I know they, they won’t do anything else. Whereas then you have those naturally athletic and energetic ones who, you’ll go outside, and you know they’ll still be playing and running around and their heart rate will get up. So if we don’t do it for them, they will still be fine.” (Implementer 4)
		Student boredom (B)	“And that’s the thing too - they get bored really quickly too.” (Implementer 8)
		Heightens awareness of physical activity importance (for student and teacher) (F)	“I think its at least started important conversations that need to happen. It has at least let all of those people- you know, students, teachers, admin- know that this is something critical that needs to be addressed and accounted for. So I think it has heightened awareness.” (Implementer 7)
		Student enjoyment is activity dependent (B,F)	“Um, my kids… yeah I mean my kids love it. They love that I would put that in a schedule. They like different activities, although they moan and groan at the different ones because they’re not interested. Um, I think kids just want to run around.” (Implementer 10)
		It’s a mental break (for student and teacher) (F)	“It’s good for the students, it’s good for me. Like, it’s umm even - like I eat my snack then too. And I actually - I want to say earlier in my career, even last year, like I used to go out and do a lap with them, just ‘cause I found for me, just the fresh air, the sunshine - if it’s sunny that day - and I would walk it as well. I used to run it. Umm but just to get moving, it helps me as well. It’s a mental break for them, it’s a mental break for me.” (Implementer 3)
		It improves students’ attention and focus which improves the learning environment (F)	“Some positive impacts for the students and teachers would be we do see more focus out of the kids after they burn off some energy. Especially the high-energy students. Um, negative effects… I think the only negative effects we would say, it would be that it… I don’t know. I don’t think there would be any. Some would complain about that it takes up time, right, out of their schedule, but I would argue then, ‘you’re getting that time back because you’re getting more quality time focus time out of the students.’” (Implementer 1) “I think it’s beneficial for all teachers because- because of the increased focus and… and their general happiness, level of happiness that just gives a more positive atmosphere in the classroom. And so that positive atmosphere - if you’ve got a positive atmosphere, kids will learn more, you know, then if they’re stressed or tired or hungry. Ya.” (Implementer 2)
Social influences	16.9	The school system prioritizes academics (B) versus they prioritize DPA (F)	“But there’s already so many other initiatives that exist in schools. Um and lots of those focus around academics. And it totally depends on your school too and what the focus is at your school. Umm because I know some places that is the focus because academically, they’re where they need to be. But for us, some of those core areas are more important at this point because we have kids who can’t read at grade levels. So for us that becomes our primary focus.” (Implementer 4) “So when I came to this elementary school that was kind of built in with their system and from what I understand other elementary schools do a similar thing, because there’s no morning recess scheduled. They kind of build in an unofficial morning recess which is the DPA and snack. So that’s what they kind of do.” (Implementer 1)
		I implement DPA just like other teachers (B,F)	“And I’m not aware of anybody in the school that’s doing it any other way. So not just do we not have a school policy, I’m not even sure if individuals - how individuals are approaching it other than what I’m doing.” (Non-implementer 1) “Ya, the other intermediate teachers I know, like I said, they are running a lap. I know that some of the primary teachers, they just go outside and do like play on the playground time. And then there’s the one class where I see the teacher walks around the school with her class. So I think that everybody’s trying to get in it, one way or another.” (Implementer 4)
		Students don’t participate and you can’t force them to move (B)	“And a lot of times they act very silly. They just think it’s funny and it just becomes something where you’re like, you know, I’ve said this before, I’m guilty of it, where it’s like ‘well if you guys aren’t going to do it, if you’re not… the purpose of DPA is to be moving the whole time. Um, that… we’re just going to pull it and we’re not going to do it.’” (Implementer 10) “You’ll see them out there and you cannot force them. That’s the challenge with DPA. I can say we are going to go out and do this. But you cannot force, make them run or whatever…I think the dilemma with DPA is that yeah, I think it’s great, but you cannot force the children to physically, to do it. They do whatever they feel like at their level.” (Implementer 8)
		A champion teacher who shares resources is helpful (F)	“If one person is willing to take on that organizational force and really bring people together and create the program, then it will happen.” (Non-implementer 1) “Every time I find something good, I will send it to other people. Like, ‘oh here’s this really cool kids yoga thing,’ or ‘here’s this really cool dance thing’ and I’ll send it along to teachers I know. Sometimes all of them. And all of the time the feedback is really good.” (Implementer 10)
		Students cue teachers verbally and non-verbally (F)	“Sometimes they say ‘can we have a break?’ I’m like ‘OK, we can do that.’” (Implementer 8) “I guess I’m just drawing judgment upon my experience and what I see. Observation. Umm are my kids wiggling in their seats, ready to go, losing focus at that time of the day? Ya, they are ready for a break. So we go, we do that break. We eat our snack. We come back in and by the time all that is said and done, they are refocused, they are ready to focus for another hour and 15 min or whatever it is. Umm and that’s why, ya I guess that’s why I do it.” (Implementer 3) “It’s usually based on the, you know, they usually cue me. They usually, you know, from their attention. That I’m like ‘Ok, we need to do something here to get them up and moving and oxygen…’ Like they just need to that - you know, a burst of oxygen in their brains to just kind of wake them up. You know what I mean? Like, ya. So, it’s basically – it’s them. I take my cues from them.” (Implementer 2)
Knowledge	8.8	DPA is not our expertise (B)	“Cause we tend to teach what we know. And PE and daily physical activity kind of sit- not with all teachers, but on the backburner of what we know really well.” (Implementer 10)
		Unaware of DPA policy requirements (B)	Although all teachers knew about the DPA policy, very few had *Knowledge* of the specific requirements relating to duration (i.e., minutes), intensity (i.e., MVPA), type (i.e., aerobic, strength, flexibility), and time of day (i.e., during instructional and non-instructional). “I think teachers don’t know enough about it-- I don’t know enough about it, and I’m pretty savvy in that area. But I couldn’t- I couldn’t tell you that I’ve actually viewed that document myself. And that’s wrong.” (Implementer 7)
Intentions	7.7	Teachers’ priorities and interests differ (B,F)	“The interest part is hard because you’re either interested or you’re not. And obviously everybody’s interests vary. So obviously that’s an interest of mine. Is it an interest of other teachers? No, they are interested in other stuff. Umm is there a way to support them? Absolutely, with stuff like that. Umm even just one person on staff going to these workshops, getting educated, getting that experience and collecting the resources and then coming back and presenting those resources to the rest of the staff. Now, that’s what I did. Now, can I boost their interest in it by doing those things? No. Can I force them to use it? No. It’s up to them after that. So it’s hard.” (Implementer 3)
		DPA is dropped for other subjects (contingent intentions) (B)	“It’s unfortunate that we kind of always push physical activity to the, you know, if we have time we’ll do it. But it is the reality of most teachers. We’re so worried about making sure that our content courses are covered, right? So that’s the biggest thing is if we’re behind schedule-wise in our class, then DPA is the one that we’re always saying, oh, we could make up another 15 min, because we already have that scheduled in. So we’ll take 15 min and not do DPA today.” (Implementer 1)
Beliefs about capabilities	5.6	DPA delivery depends on confidence and comfort-level (B,F)	“I think some of them might feel that, I mean, if they don’t exercise, or they don’t, they’re not knowledgeable about healthy habits in their own life- ‘cause lots of people aren’t knowledgeable- that they wouldn’t want to model it anyways in school. So those would probably be the teachers- Not that they wouldn’t do it, but they would put on DVDs or you know, play games or something like that. Um, I don’t know why they wouldn’t be confident. I think that would probably be my biggest thing. It’s when… even in other subjects, if I’m not confident in teaching - French is another one - um, that somebody might not be confident in, that they wouldn’t spend a lot of time on French.” (Implementer 10) “Like, if you ask that question maybe for someone else who didn’t feel as comfortable teaching physical activity, PE, they tend to not do as much.” (Implementer 1)
		It’s difficult to motivate students (B)	Teachers discussed that it was not the provision of DPA opportunities that was necessarily difficult, but the motivation of children who were not motivated to be active. “Do I wish I could find some way to motivate those kids to do that? Absolutely… it’s hard as a teacher to motivate those kids that don’t even want to participate.” (Implementer 3)
		It’s easy to implement (F)	“But I just don’t think it’s very difficult to implement.” (Implementer 10)
Skills	4.3	Initial DPA-specific training was good but insufficient/inappropriate over time (B,F)	“When this all first came into play, we did ActionSchools. So that was our day. We had a specialist come. We tried out a bunch of the games. We opened up the bins. We looked at what kind of resources there were. And then we did some kind of team building, brain storming, ‘what could this look like in your classroom?’ It was a great day. Um that was the only support that we were given.” (Implementer 7)
		Previous training and experience is helpful (F)	“I would say I do [have the necessary skills] because I did my entire undergrad in Human Kinetics, in Exercise Science. And obviously when I went through that program, we did do a lot - I don’t want to say a lot - but we did do some PE-related courses. So, did I take a soccer course, a basketball- I took all that stuff and obviously learnt about the benefits of it all. But I think just with my background in it, I am probably more well equipped as a teacher umm to just - I can seriously just take my kids out and wing a game and I just know how to do it because I’ve done it so many times.” (Implementer 3)
Social/professional role and identity	3.3	It’s not my job/responsibility (B)	“We are - I felt my job is an educator and we seem to be taking on a lot of society’s jobs. Family’s jobs. And I thought, you know, I’m kind of up to here with the responsibility for every little thing. That was my - definitely my first thought.” (Non-implementer 1) “I mean, at first I was sort of like, ‘well, we’re doing parental jobs now? Like is it not the parents job to ensure that their child is…’ And I still think that. I think it’s up to a parent to make sure they’re providing their kid with opportunities at home. Umm I do believe that. And if there’s a day that we don’t do DPA and the kids complain, I’m like ‘well run home. What do you do when you go home? Play with your video game?’ You know? So I don’t really feel too bad if we don’t get to it or if it’s a day where it’s only 15 min. Umm so I do think there is a responsibility in the home to ensure that your child is getting some exercise, for sure. Ya.” (Implementer 5)
		It’s my professional obligation (F)	“For me, as with anything else on my report card, like I have to know that I covered it, that they did it and that I evaluated it appropriately. I have to know that as an educator myself. That’s a professional standard that I hold myself.” (Implementer 7)
		It’s important to me because I’m active (F)	“I’m interested in maintaining a good physical health in my own life, it just plays into my teaching because of my identity.” (Implementer 10) “But like, I’m passionate about sports. I’ve done sports my whole life and I’m always coaching. Like I’m the coach here…and I probably do our DPA more consistently than others.” (Implementer 1)
Reinforcement	2.8	Lack of monitoring (B)	No teachers said that they were assessed on whether or not they implement DPA. While they are required to report children’s fulfillment of DPA on the report cards (i.e., ‘meeting’, or ‘not meeting’), most teachers believed this system had no effect on the implementation of the policy. “It’s kind of like having a law, right? If you have a law in place on paper, that’s all good. But if it’s never enforced from your law officials, right? Then no one’s ever going to take… they’re not going to put any stock into it. So that’s how I feel right now. It’s not really… ‘enforce’ is a bad way of putting it, but, yeah. It’s never monitored I guess.” (Implementer 1)
Optimism	2.8	Optimism depends on student’s motivation to be active (B,F)	Teachers had mixed feelings about the success of the DPA policy, linking their optimism to student’s motivation to be active. “You’re going to have someone in that group that does not want to do that. That doesn’t like it. And so, you can’t force them, you know? Our hands are tied. So the whole DPA is an awesome idea, but it’s not practical if the kid doesn’t want to do it. They’re not going to do it. So you just try to do it as much as you can and get them to participate as much as they’re willing to.” (Implementer 8) “…the kids that are going to be active, are going to be active. And the kids that aren’t, aren’t.” (Non-implementer 3)
Emotion	2.4	It’s frustrating (B)	“I don’t think its just frustration around DPA, but it’s frustration around finding the time to accomplish all the expectations. And it’s not horrible because I mean, I absolutely love my job and I wouldn’t want to do anything else. … So I don’t think it’s just DPA, I think it’s just the rigors of it all.” (Non-implementer 2)
		I’m worried that students will get hurt (B)	“But I mean because I’m not trained in that kind of stuff, it does worry me sometimes that I’m doing the activities that are by trained people and then, you know I’m a smart person, so I know about injury and I know about warming up and that kind of stuff but I’m not an expert. So what would happen if a kid pulled a muscle really badly or something and their parents… their parents probably could get angry and I could get in some sort of trouble. So that’s a worry of mine. I guess it’s a, it would be a restriction.” (Implementer 10)
		It’s a joke (B)	“When you brought it up of course I was smiling or if not a smirk, cause it’s joke-like. How do you fit it in a day already that’s overscheduled.” (Implementer 6)
		I enjoy it too (F)	“And it’s funny – you’d think that it’s for the kids but I use it a lot for myself too. If I can get a stretch in there, I’m feeling better for the rest of the day.” (Implementer 6) “And I love doing it. I look forward to that movement break.” (Implementer 7)
Goals	1.9	Planning for and scheduling DPA into the timetable (F)	“I think it definitely has to do with having a set schedule that’s working now, and this is now the third year of kind of this type of schedule that I’ve been using. So like I said, first year was my first year in elementary school here and then second year was kind of, I did a similar schedule and then I changed everything around and actually built in DPA in these blocks. So I think that’s the major drive behind it.” (Implementer 1)
Memory, attention and decision processes	0.01	Forgetting about DPA (B)	Overall, almost all teachers discussed not remembering or forgetting to implement DPA during the school day.
		I don’t think about it, it’s a routine (F)	“I think initially when we first started it, I was very conscientious about that but now I think I don’t really think about it, we just kinda, incorporate it.” (Implementer 5) “I want to say it’s just routine. Like I write out a day plan every single day. And every single day I just write it in there. And actually, I plan the blocks I have before and I plan the blocks after for that umm it’s -- and I know it’s not a specific time in here. But- and in my mind I’m always like it’s 15 min. But it’s not. It turns into 20–25 min pretty much everyday. But I plan accordingly. And it’s a routine, it’s something we do everyday.” (Implementer 3)
Behavioural regulation	0.007	Writing it down (on timetable, board) helps us remember (F)	Teachers who implemented DPA regularly discussed the importance of writing it on their timetable or the board in order to remind themselves to conduct DPA. “I do that [schedule it in] because that’s just - well, that shows my thinking and it’s my plan. And then ugh, it reminds me to do it, or that kind of thing. Umm, and then I also put it on the board, right? Because everyday the agendas up.” (Implementer 5)

B, barrier; F, facilitator

All quotes are in parentheses and clarifying text is not

Note: In this table, implementer refers to instructional implementer and non-implementer refers to non-instructional implementer

### Comparison of barriers and facilitators by teacher implementation approach

Teachers, irrespective of implementation approach (i.e., whether or not they provided DPA during the instructional school day) experienced similar barriers and facilitators with regards to *Skills* (e.g., DPA-specific training, previous training/experience), *Knowledge* (e.g., lack of knowledge about DPA requirements), *Environmental context and resources* (e.g., poor, inappropriate or lack of DPA-specific training; lack of time due to curricular demands and schedule interruptions; weather and space constraints), *Reinforcement* (e.g., lack of monitoring), *Social influences* (e.g., school-level priorities, support from other teachers, student participation), and *Optimism* (e.g., mixed feelings about success of policy).

Where teachers who implemented DPA during instructional time differed from those who relied on non-instructional time was in their *Goals* and *Behavioural regulation* (e.g., planning for and scheduling DPA in timetable; providing schedule to students), *Social/professional role and identity* (e.g., strong personal physical activity identity and belief in responsibility to get children active at school), experience of the *Consequences* (e.g., linking physical activity to improvements in attention and focus for a better classroom learning environment) and *Social influences* (e.g., recognizing and responding to children’s verbal and non-verbal cues to move throughout the day).

## Discussion

Similar to the review examining the barriers and facilitators to DPA policy implementation in Canada [42], this study highlights teachers’ implementation of the DPA policy may be impacted by factors relating to *ECR*, *Beliefs about consequences*, and *Social influences,* as well as *Knowledge* and *Intentions*. The identified themes in this study have been reported in other DPA studies [17–21, 43–47], as well as studies examining the implementation of other school-based PA initiatives [48–52]. For example, similar themes in the *ECR* domain include lack of time in the schedule due to competing curricular demands [17–19, 21, 43–46, 50–52], access to resources (space, facilities, equipment and ideas) [17–19, 21, 43–50], and inclement weather [43–46]. Related *Beliefs about consequences* themes include an increase in teacher workload, burden and stress [18, 44, 46], improved student focus, attention and/or academic performance [17, 18, 44, 46, 49, 51], improved classroom learning environment [17–19, 46], and overall student enjoyment and interest in physical activity [17, 44, 46]. Similar themes within the *Social influences* domain include level of support from staff, administration and other school champions [18, 20, 21, 44, 48, 49], and student participation/preferences [18, 20, 21, 45, 48]. Due to these similarities, it is possible that intervention designs based on this study may be effective within other school contexts (e.g., different provinces/countries).

Addressing barriers to implementation is important because these factors affect implementation fidelity, which in turn has implications on the policy meeting its desired outcomes. Very little research has examined the impact of these policies on children’s physical activity levels at school [16, 42]. Considering the different approaches to implementation by teachers in this study, it is possible the different approaches result in different outcomes. This study compares similarities and differences in perceived barriers and facilitators to DPA implementation by teacher-reported implementation approach, suggesting that a targeted intervention approach is necessary for different contexts. Future studies should examine effectiveness of these approaches on physical activity levels of children at school through objective measurement. This study’s findings can be used to provide context for and interpret why different DPA policy implementation approaches succeed or fail to meet intended outcomes at the student level [53].

While there are added challenges to the provision of DPA opportunities during instructional time (as opposed to relying on non-instructional time for children to be active), the instructional implementers were able to overcome these challenges. Common challenges reported by both instructional and non-instructional implementers included issues relating to *ECR* (e.g., lack of time, resources and space) and *Social influences* (e.g., lack of school-level priority). It may be that teachers who implement DPA during instructional time are better able to overcome these underlying organizational barriers to DPA delivery. Accordingly, instructional teachers differed from non-instructional teachers on a number of behavioural domains, particularly those in which they could exert a degree of individual control, such as planning, scheduling and having strong personal beliefs in the importance of physical activity. For example, instructional implementers discussed facilitators with regards to *Goals* and *Behavioural regulation* (e.g., planning for and scheduling DPA in the timetable), and in their *Social/professional role and identity* (e.g., strong personal PA identity and belief in their responsibility to get children active at school). Non-instructional teachers did not plan for (i.e., set goals) or schedule DPA into their timetables (i.e., regulate their provision of DPA), both of which helped to facilitate instructional implementers provision of DPA opportunities during the instructional school day. Therefore, while it may seem that targeting barriers to DPA implementation may provide an effective means to improve implementation, an important distinction may be the factors that assist the instructional teachers in providing more DPA opportunities during instructional time. Researchers may want to consider these variations for intervention design and delivery in specific contexts.

### Implications

The current study builds on previous research examining the factors influencing the implementation of DPA in Canada through the inclusion of an evidence-based determinant framework by which to provide a theory-based analysis of the implementation barriers and facilitators. Embedding these factors within the TDF domains enables researchers to develop interventions aimed at targeting the constructs shown to have the most salient influence on behaviour. This behavioural diagnosis is also relevant to policy makers who wish to better support teachers in their implementation efforts. In this study, teachers most often discussed factors within the *ECR*, *Beliefs about consequences*, *Social influences, Knowledge* and *Intentions* domains. When organized heuristically, these domains are representative of all sources of behaviour in the COM-B model, namely capability (*Knowledge*), opportunity (*ECR*, *Social influences*) and motivation (*Beliefs about consequences, Intentions*) components, and have important implications for theory selection in intervention design. These findings suggest that all components are interacting to influence teachers’ DPA implementation behaviours, and therefore selecting a theory that broadly encompasses all determinants of behaviour may be more successful at promoting behavior change. Alternatively, reflective motivation theories may not be the most effective option for intervention design because they fail to consider the broader physical and social-environmental influences on behaviour. Findings from other DPA studies in Canada have found that both individual- and organizational-level factors influence DPA implementation. In Ontario, for example, Allison and colleagues [43] found that policy awareness, teacher self-efficacy, scheduling and monitoring are significant predictors of implementation fidelity. Efforts to improve implementation must target these individual- and system-level factors.

To create interventions, the relevant theoretical domains can be mapped onto intervention functions (e.g., via the Behaviour Change Wheel framework [BCW]) [26] and behaviour change techniques [23]. For example, possible intervention functions to target *ECR* include Training, Restriction, Environmental restructuring and Enablement. To minimize teachers’ perception of a lack of time (due to curricular demands), an intervention could be designed to *train* teachers how to incorporate physical activity into other lessons. Likewise, consideration of competing behaviours, namely other school curriculum subjects, may be another means by which to minimize the burden of a lack of time. As another example, the education intervention function could be used to target the teachers’ lack of knowledge of DPA policy guidelines, and could be delivered by improving policy guideline dissemination and providing clear recommendations to teachers on how to achieve these guidelines.

After using the TDF to understand the behaviour, intervention designers can select the behaviour change techniques (BCTs), or active intervention components, aimed at targeting the relevant domains. For instance, BCTs that have been mapped to the ECR domain include: restructuring the physical or social environment, discriminative (learned) cue, prompts/cues, or avoidance/changing exposure to cues for the behaviour [54]. To address the lack of time example provided above, schools could *restructure the environment* by creating policies whereby teachers must schedule opportunities for their students to be active into their timetables. However, adoption of individual school policy would first require considerable changes to overcome factors working at the social- and structural-level. Ultimately, final decisions about intervention functions, BCTs and modes of delivery can only be selected according to what can be feasibly and acceptably delivered within the specific school context [23].

### Strengths and limitations

The main strength of this study was the use of the Theoretical Domains Framework to categorize and comprehend implementation barriers and facilitators. However, the TDF is not a theory, and therefore it cannot provide an explanation as to how these domains are connected and influence one another [53], limiting our understanding of how these factors interact in complex contexts. While the TDF showed good utility for categorizing barriers and facilitators within this context, it was difficult to differentiate between some domains (e.g., *Beliefs about consequences* and *Optimism*), noted too by other researchers [35, 37]. Additionally, using the TDF framework to guide the interview schedule and deductively code barriers and facilitators means that the researchers approached the data with an informed, yet potentially strong bias. However, the interview protocol was designed to minimize leading questions and extracting barriers and facilitators prior to coding into specific domains was done to minimize bias of identification of relevant text and increase trustworthiness. Although the interviewer asked participants to provide examples of barriers/facilitators that they had experienced versus perceived to impact DPA implementation, and efforts were made to minimize hypothetical barrier/facilitator extraction, it is possible that this distinction was not clearly discernable for participants. According to Sparkes and Smith [55], a general weakness of content analyses is that they suggest that the more themes or categories that are counted reflect the meaningfulness or significance of that category. In this study, the total count (n) coded to each TDF domain included repeated barriers/facilitators and each count reflect the proportion of time that the teachers dedicated to discuss the respective factor. Therefore, frequency of barriers/facilitators coded to domains should not be a proxy for importance or significance. Some domains or themes that occurred only a few times may be highly meaningful to a teachers’ implementation of DPA and thus be areas of potential interest (and future research) for those creating interventions to target these factors in the future. Finally, this study aimed to include teachers with diverse DPA implementation approaches. Unfortunately, it was difficult to identify and recruit teachers who did not provide DPA opportunities during instructional time, most likely due to social desirability bias.

## Conclusion

Given that the effectiveness of school-based physical activity policies depends on their implementation, it is important to understand the challenges that teachers face in providing physical activity opportunities at school and to identify the levers that increase implementation. This study theoretically identified the barriers and facilitators impacting the implementation of the DPA policy in British Columbia and this information can be used to explain how the context influences the success or failure of the policy. The advantage of using a theoretical framework to understand the barriers is that it can assist researchers in the systematic development of future interventions to target the factors shown to impede implementation.

## Additional files

Additional file 1: TDF domain definitions. TDF with domain definitions (DOCX 117 kb)

Additional file 2: Consolidated criteria for reporting qualitative research (COREQ). 32-item reporting checklist for qualitative research (DOCX 20 kb)

Additional file 3: Interview guide. Interview guide organized by TDF domain (DOCX 120 kb)

Additional file 4: TDF coding manual. TDF domain definitions and coding notes used to code barriers and facilitators (DOCX 100 kb)

Additional file 5: Double extraction agreement. Inter-coder percent agreement across four barrier and facilitator extraction rounds (DOCX 40 kb)

Additional file 6: Ineligible extracted barriers and facilitators. Total counts of extracted barriers and facilitators that were ineligible or uncodable (DOCX 49 kb)

Additional file 7: Inter-coder agreement statistics. Inter-coder agreement statistics including percent agreement, Kappa and PABAK and the number of observations used during each coding round (DOCX 72 kb)

Additional file 8: Barriers and facilitators by TDF domain and implementation approach. Frequency counts of barriers and facilitators coded to each TDF domain by teacher implementation approach (DOCX 77 kb)

## Abbreviations

DPA: Daily Physical Activity policy
ECR: Environmental context and resources (TDF domain)
PABAK: Prevalence adjusted bias adjusted Kappa statistic
TDF: Theoretical Domains Framework

## Footnotes

1 Over the course of the planning for and data collection of this study, the BC Ministry of Education revised the DPA policy statement multiple times. As such, there is no longer any division between PE and non-PE days. The DPA requirements of 30 min of physical activity are now required irrespective of school days with or without PE.
