# Supplementary material for: Barriers and facilitators to the implementation of a school-based physical activity policy in Canada: application of the theoretical domains framework
Source: BMC Public Health. 2017 Oct 23;17:835. doi: 10.1186/s12889-017-4846-y (PMC5654002; doi:10.1186/s12889-017-4846-y)
Supplement: Supplementary file 1 — TDF domain definitions. TDF with domain definitions (DOCX 117 kb) [file 12889_2017_4846_MOESM1_ESM.docx]

**Additional file 1. TDF domain definitions**

| **TDF Domain** | **Definition** |
| --- | --- |
| Skills (physical, cognitive and interpersonal) | An ability or proficiency acquired through practice |
|  |  |
| Knowledge | An awareness of the existence of something |
| Memory, attention and decision processes | The ability to retain information, focus selectively on aspects of the environment and choose between two or more alternatives |
| Behavioural regulation | Anything aimed at managing or changing objectively observed or measured actions |
| Social/professional role and identity | A coherent set of behaviours and displayed personal qualities of an individual in a social or work setting |
| Beliefs about capabilities | Acceptance of the truth, reality, or validity about an ability, talent, or facility that a person can put to constructive use |
| Optimism | The confidence that things will happen for the best or that desired goals will be attained |
| Beliefs about consequences | Acceptance of the truth, reality, or validity about outcomes of a behaviour in a given situation |
| Intentions | A conscious decision to perform a behaviour or a resolve to act in a certain way |
| Goals | Mental representations of outcomes or end states that an individual wants to achieve |
| Reinforcement | Increasing the probability of a response by arranging a dependent relationship, or contingency, between the response and a given stimulus |
| Emotion | A complex reaction pattern, involving experiential, behavioural, and physiological elements, by which the individual attempts to deal with a personally significant matter or event |
| Environmental context and resources | Any circumstance of a person's situation or environment that discourages or encourages the development of skills and abilities, independence, social competence, and adaptive behaviour |
| Social influences | Those interpersonal processes that can cause individuals to change their thoughts, feelings, or behaviours |

Definitions taken from Cane, O’Connor & Michie (2012). TDF, Theoretical Domains Framework
