# Supplementary material for: Barriers and facilitators to the implementation of a school-based physical activity policy in Canada: application of the theoretical domains framework
Source: BMC Public Health. 2017 Oct 23;17:835. doi: 10.1186/s12889-017-4846-y (PMC5654002; doi:10.1186/s12889-017-4846-y)
Supplement: Supplementary file 3 — Interview guide. Interview guide organized by TDF domain (DOCX 120 kb) [file 12889_2017_4846_MOESM3_ESM.docx]

**Additional file 3. Interview guide**

Date: _________________________

Participant ID: __________________

Interviewer: ____________________

- Remind participant to bring completed timetable for week prior to interview

1. Thank participant for their time. Establish ‘cultural ignorance’ and remind them that you are not going to judge any of their thoughts or feelings. There are no right or wrong responses to these questions.
2. Remind participant about confidentiality and right to withdrawal from question/study.
3. Obtain permission to record interview.
4. Start interview:

| **Purpose** | **Question** |
| --- | --- |
| Participant Demographics | What grade do you currently teach?  How many years of experience do you have teaching in elementary school? (all in BC? Prior to 2008) |
| Knowledge (TDF) | Before this study, were you aware of the current DPA initiative’s requirement for daily physical activity at school for children?  What is your understanding of the expectations of the DPA policy? Can you please explain your understanding of the DPA policy?  How does this policy relate to the physical activity guidelines for children aged 5-13? What are they? |
| Implementation Strategies | From my understanding, there are no specific guidelines from the Ministry of Education on how to implement DPA, so I am interested in finding out more about how BC schools are meeting these requirements.  How is the DPA policy implemented at your school? Does your school provide any guidelines on how to do so?  How do you implement the DPA policy in your classroom?  What is your most common strategy to implement DPA in your classroom?  Why do you choose some activities over others?  How do you ensure that you implement DPA?  How do you try to ensure that the children are working at a moderate and/or vigorous intensity? |
| Broad Questions re: Implementation factors | Are there any factors that affect if or how you implement DPA in your classroom/school? If so, what?  Are there situations that make it difficult to conduct DPA?  Is it difficult to get the children active at moderate and vigorous intensities? |
| Goals (TDF) | Are there any strategies you have put in place to ensure you could implement the DPA policy in your classroom?  Do you make a plan as to how you implement the DPA policy in your  classroom? Do you follow it? |
| Skills (TDF) | Do you feel as though you had the necessary skills and training to implement DPA in your classroom? (put it in context relating to working prior to 2008 if applicable)  Were you provided with additional training to implement DPA?  Have you received additional support materials to implement DPA (e.g. game ideas, equipment, funding)? Please provide examples. |
| Beliefs about capabilities (TDF) | Do you think that implementation of DPA has anything to do with confidence- from the teachers, children, parents, etc.?  Do you feel confident educating and encouraging students to engage in physical activity?  Do you feel confident implementing DPA?  What would help you feel more confident to implement DPA? |
| Behavioural Regulation/ Reinforcement (TDF) | Are any methods being used to assess accountability to DPA implementation by the schools or teachers? (E.g., report cards, monitoring school compliance/success of the DPA policy)?  Does your school principal or administration check to ensure DPA is being conducted in your classroom?  What role/effect do you think the DPA grading system in report cards has had on the implementation of this policy?  How do you think DPA should be monitored in schools/classrooms?  Are you rewarded or reprimanded for (not) implementing DPA? |
| Intentions (TDF) | At the outset of the program/of the week, did you intend to implement the DPA policy?  (Since inception), has your willingness to implement DPA changed over time (more or less)?  Has your motivation changed about doing DPA over the course of your teaching experience? |
| Memory, Attention and Decision Processes (TDF) | Do you sometimes forget to implement DPA in your classroom?  Are there any situations that make it difficult for you to remember to implement DPA? |
| Environmental Context and Resources (TDF) | What factors influence whether or not you implement DPA?  Are there any competing tasks or time constraints that influence your decision to implement DPA? If so, what?  Do you have all the materials and equipment you need to implement DPA in your classroom?  Have you received implementation support in terms of program support materials and training? |
| Social Influences (TDF) | Are other teachers in your school implementing DPA?  Do you feel supported by other teachers/principal/administration/school district to implement the DPA policy?  Is there anyone you would want to receive support from? |
| Social/Professional Role and Identity (TDF) | Does the DPA policy align with you personal PA beliefs and/or teaching philosophy?  In general, do you think of yourself as someone who engages in physical activity? Maintains a healthy diet?  Did you feel as though you had ownership of how you conduct DPA?  Do you see any ethical, legal or social issues with implementing the program? |
| Optimism (TDF) | Do you feel that the DPA policy will be successful? |
| Beliefs about Consequences (TDF); Perceived outcomes | What positive or negative impact do you think the DPA policy has had on the school community (school, teachers, students, and parents)?  Do you think that children are more physically active at school when they engage in DPA compared to when they do not? Or compared to when they are in PE class? |
| Emotions (TDF) | What are your feelings about conducting DPA? (For example: scared, worried, excited) |
| Final Questions | Do you have any feedback on how to improve the DPA policy?  Is there anything else you would like to add about your experience as a teacher implementing DPA in your classroom? |
