# Supplementary material for: Barriers and facilitators to the implementation of a school-based physical activity policy in Canada: application of the theoretical domains framework
Source: BMC Public Health. 2017 Oct 23;17:835. doi: 10.1186/s12889-017-4846-y (PMC5654002; doi:10.1186/s12889-017-4846-y)
Supplement: Supplementary file 4 — TDF coding manual. TDF domain definitions and coding notes used to code barriers and facilitators (DOCX 100 kb) [file 12889_2017_4846_MOESM4_ESM.docx]

**Additional file 4. TDF coding manual**

| **TDF Domain** | **Definition** | **Notes (Themes)** |
| --- | --- | --- |
| Skills (physical, cognitive and interpersonal) | An ability or proficiency acquired through practice | - (Lack of) DPA-specific training* - Previous experience/training |
|  |  |  |
| Knowledge | An awareness of the existence of something | - (Lack of) DPA-specific training* - Previous experience/training |
| Memory, attention and decision processes | The ability to retain information, focus selectively on aspects of the environment and choose between two or more alternatives | - Forget/remember about DPA |
| Behavioural regulation | Anything aimed at managing or changing objectively observed or measured actions | - Self-monitoring (e.g., tracking DPA implementation) |
| Social/professional role and identity | A coherent set of behaviours and displayed personal qualities of an individual in a social or work setting | - Personal physical activity beliefs/identity - (Not) teacher’s role/responsibility |
| Beliefs about capabilities | Acceptance of the truth, reality, or validity about an ability, talent, or facility that a person can put to constructive use | - Ease or difficulty of implementation - Comfort/energy/confidence level |
| Optimism | The confidence that things will happen for the best or that desired goals will be attained | - (No) effect on overall PA levels |
| Beliefs about consequences | Acceptance of the truth, reality, or validity about outcomes of a behaviour in a given situation | - Positive: teachers/children aware of PA as important/beneficial, child enjoyment/fun; impact on child focus/learning; increases on children’s PA levels or participation - Negative: takes time away from teaching; no effect on children’s PA levels or participation, injury/pain (due to poor student skill/ability), student frustration/boredom |
| Intentions | A conscious decision to perform a behaviour or a resolve to act in a certain way | - Priority/value/(self) pressure at individual level - General motivation - Deciding to drop DPA for other subjects (“Contingent intentions”) - Trying/effort |
| Goals | Mental representations of outcomes or end states that an individual wants to achieve | - Plan/schedule DPA on timetable, writing DPA on board |
| Reinforcement | Increasing the probability of a response by arranging a dependent relationship, or contingency, between the response and a given stimulus | - Perception of (lack of) monitoring |
| Emotion | A complex reaction pattern, involving experiential, behavioural, and physiological elements, by which the individual attempts to deal with a personally significant matter or event | - Personal fun/enjoyment - Frustration |
| Environmental context and resources | Any circumstance of a person's situation or environment that discourages or encourages the development of skills and abilities, independence, social competence, and adaptive behaviour | - (Lack of) DPA-specific training* - Poor/inappropriate training - Autonomy supportive (e.g., flexibility to conduct DPA whenever) - Curriculum demands - Weather/time of year - Lack of time - Schedule interruptions (e.g., assemblies, field trips) - Space - Equipment - Ideas |
| Social influences | Those interpersonal processes that can cause individuals to change their thoughts, feelings, or behaviours | - Supportive others (teachers, administration, principal) - Mentorship (i.e., sharing resources; champion) - Priority/value at group level (e.g., whole-school approach/school culture) - Pressure to focus on other subjects - Generic support (i.e., no resources specified) - (Dis)similar others - School recommendations/ specifications on DPA implementation - Students** – injury/illness/ tiredness; needs to move/cues; motivation/preferences/ participation |

Coding manual based on definitions provided in Cane, O’Connor & Michie (2012). TDF, Theoretical Domains Framework

*A general lack of training was always coded under Skills AND Knowledge AND ECR

** Students influence teacher’s implementation of DPA for multiple reasons: injury, cues (seeing that the children are not focused, asking for DPA break)
