# Supplementary material for: Barriers and facilitators to the implementation of a school-based physical activity policy in Canada: application of the theoretical domains framework
Source: BMC Public Health. 2017 Oct 23;17:835. doi: 10.1186/s12889-017-4846-y (PMC5654002; doi:10.1186/s12889-017-4846-y)
Supplement: Supplementary file 5 — Double extraction agreement. Inter-coder percent agreement across four barrier and facilitator extraction rounds (DOCX 40 kb) [file 12889_2017_4846_MOESM5_ESM.docx]

**Additional file 5. Double extraction agreement**

| Round | *n* barriers | *n* facilitators | *n* total | Total mean percent positive agreement |
| --- | --- | --- | --- | --- |
| 1 | 40 | 30 | 70 | 85.7 |
| 2 | 38 | 44 | 82 | 85.4 |
| 3 | 30 | 66 | 96 | 86.5 |
| 4 | 55 | 40 | 95 | 87.4 |
| Total (average on all) | 163 | 180 | 343 | 86.3 |
