# Supplementary material for: Barriers and facilitators to the implementation of a school-based physical activity policy in Canada: application of the theoretical domains framework
Source: BMC Public Health. 2017 Oct 23;17:835. doi: 10.1186/s12889-017-4846-y (PMC5654002; doi:10.1186/s12889-017-4846-y)
Supplement: Supplementary file 6 — Ineligible extracted barriers and facilitators. Total counts of extracted barriers and facilitators that were ineligible or uncodable (DOCX 49 kb) [file 12889_2017_4846_MOESM6_ESM.docx]

**Additional file 6. Ineligible extracted barriers and facilitators**

| Round | *n* barriers | *n* facilitators | *n* total |
| --- | --- | --- | --- |
| Round 1 | 0 | 0 | 0 |
| Round 2 | 1 | 1 | 2 |
| Round 3 | 2 | 0 | 1+1 |
| Round 4 | 2 | 0 | 2 |
| Round 5 | 2 | 3 | 5 |
| Round 6 | 2 | 2 | 4 |
| Round 7 | 2 | 4 | 6 |
| Round 8 | 4 | 9 | 13 |
| Round 9 | 1 | 8 | 9 |
| Round 10 | 8 | 5 | 13 |
| Round 11 | 1 | 3 | 4 |
| Round 12 | 1 | 7 | 8 |
| **Total** | **26** | **42** | **68** |

*Factors were deemed ineligible mostly due to them being hypothetical, or not affecting the targeted behaviour, namely teacher’s provision of DPA opportunities during instructional time

** Items were deemed uncodable if they were too vague to be certain which domain the factor fit under
