# Supplementary material for: Barriers and facilitators to the implementation of a school-based physical activity policy in Canada: application of the theoretical domains framework
Source: BMC Public Health. 2017 Oct 23;17:835. doi: 10.1186/s12889-017-4846-y (PMC5654002; doi:10.1186/s12889-017-4846-y)
Supplement: Supplementary file 7 — Inter-coder agreement statistics. Inter-coder agreement statistics including percent agreement, Kappa and PABAK and the number of observations used during each coding round (DOCX 72 kb) [file 12889_2017_4846_MOESM7_ESM.docx]

**Additional file 7. Inter-coder agreement statistics**

| Round | *n* barriers | *n* facilitators | % total (*n* observations) | Mean percent positive agreement (*n* observations*) | Mean Kappa (±SD) | Mean PABAK (±SD) |
| --- | --- | --- | --- | --- | --- | --- |
| Round 1 | 44 | 32 | 9.1 (76) | 35.3 (139) | 0.47 ± 0.49 | 0.84 ± 0.14 |
| Round 2 | 10 | 48 | 7.0 (58) | 61.8 (89) | 0.75 ± 0.41 | 0.92 ± 0.13 |
| Round 3 | 37 | 43 | 9.6 (80) | 63.1 (141) | 0.76 ± 0.34 | 0.91 ± 0.12 |
| Round 4 | 38 | 33 | 8.5 (71) | 63.7 (113) | 0.79 ± 0.34 | 0.92 ± 0.12 |
| Round 5 | 18 | 15 | 4.0 (33) | 75.0 (48) | 0.87 ± 0.29 | 0.95 ± 0.10 |
| Round 6 | 21 | 43 | 7.7 (64) | 65.2 (112) | 0.79 ± 0.30 | 0.92 ± 0.12 |
| Round 7 | 39 | 32 | 8.5 (71) | 66.3 (104) | 0.79 ± 0.36 | 0.93 ± 0.11 |
| Round 8 | 49 | 30 | 9.5 (79) | 61.3 (137) | 0.78 ± 0.34 | 0.91 ± 0.13 |
| Round 9 | 29 | 54 | 10.0 (83) | 55.2 (154) | 0.70 ± 0.41 | 0.89 ± 0.15 |
| Round 10 | 46 | 49 | 11.4 (95) | 54.3 (173) | 0.70 ± 0.35 | 0.89 ± 0.12 |
| Round 11 | 22 | 39 | 7.3 (61) | 62.5 (96) | 0.75 ± 0.38 | 0.92 ± 0.12 |
| Round 12 | 38 | 23 | 7.3 (61) | 52.6 (116) | 0.66 ± 0.39 | 0.88 ± 0.14 |
| **Total (across all rounds)** | **391** | **441** | **100.0 (832)** | **59.7 (1422)** | **0.73 ± 0.37** | **0.91 ± 0.13** |

*Some factors were coded under multiple domains if applicable. Mean percent was calculated based on each code the B/F item was given.
