# Supplementary material for: Barriers and facilitators to the implementation of a school-based physical activity policy in Canada: application of the theoretical domains framework
Source: BMC Public Health. 2017 Oct 23;17:835. doi: 10.1186/s12889-017-4846-y (PMC5654002; doi:10.1186/s12889-017-4846-y)
Supplement: Supplementary file 8 — Barriers and facilitators by TDF domain and implementation approach. Frequency counts of barriers and facilitators coded to each TDF domain by teacher implementation approach (DOCX 77 kb) [file 12889_2017_4846_MOESM8_ESM.docx]

Additional file 8. Barriers and facilitators by TDF domain and implementation approach

|  | **Instructional (*n* = 10)** | | **Non-instructional (*n* = 3)** | | **Total (*n* = 13)** | |
| --- | --- | --- | --- | --- | --- | --- |
| **TDF Domain** | ***n* barriers** | ***n* facilitators** | ***n* barriers** | ***n* facilitators** | ***n* total^1^** | **% total^1^** |
| Skills | 12 | 31 | 2 | 4 | 49 | 4.3 |
| Knowledge | 38 | 51 | 6 | 5 | 100 | 8.8 |
| Memory, attention and decision processes | 7 | 3 | 1 | 0 | 11 | 0.01 |
| Behavioural regulation | 0 | 8 | 0 | 0 | 8 | 0.007 |
| Social/professional role and identity | 7 | 25 | 2 | 4 | 38 | 3.3 |
| Beliefs about capabilities | 25 | 26 | 8 | 5 | 64 | 5.6 |
| Optimism | 15 | 11 | 3 | 3 | 32 | 2.8 |
| Beliefs about consequences | 61 | 147 | 9 | 8 | 225 | 19.7 |
| Intentions | 34 | 41 | 8 | 5 | 88 | 7.7 |
| Goals | 1 | 20 | 1 | 0 | 22 | 1.9 |
| Reinforcement | 22 | 4 | 4 | 2 | 32 | 2.8 |
| Emotion | 13 | 10 | 4 | 0 | 27 | 2.4 |
| Environmental context and resources | 133 | 69 | 33 | 15 | 250 | 21.9 |
| Social influences | 61 | 111 | 8 | 13 | 193 | 16.9 |
| Other | 2 | 0 | 0 | 0 | 2 | 0.002 |
| **Total** | **431** | **557** | **89** | **64** | **1141** | **100** |

TDF, Theoretical Domains Framework

^1^ The total (*n* and %) coded to each TDF domain represents the proportion of interview time across the sample spent discussing barriers and facilitators within each domain
